# Role-Play-Based Guidance for Job Interviews Using an Android Robot for Individuals With Autism Spectrum Disorders

**DOI:** 10.3389/fpsyt.2019.00239

**Published:** 2019-04-11

**Authors:** Hirokazu Kumazaki, Taro Muramatsu, Yuichiro Yoshikawa, Yoshio Matsumoto, Hiroshi Ishiguro, Masaru Mimura, Mitsuru Kikuchi

**Affiliations:** ^1^Department of Clinical Research on Social Recognition and Memory, Research Center for Child Mental Development, Kanazawa University, Kanazawa, Japan; ^2^Department of Neuropsychiatry, Keio University School of Medicine, Tokyo, Japan; ^3^Department of Systems Innovation, Graduate School of Engineering Science, Osaka University, Osaka, Japan; ^4^JST ERATO ISHIGURO Symbiotic Human-Robot Interaction, Osaka, Japan; ^5^Service Robotics Research Group, Intelligent Systems Institute, National Institute of Advanced Industrial Science and Technology, Tsukuba, Japan

**Keywords:** autism spectrum disorders, android robot, job interview, role-play, self-confidence

## Abstract

Interventions for job interviews targeting the impaired theory of mind observed in individuals with autism spectrum disorders (ASD) are limited. We developed a role-play-based guidance system for job interviews using an android robot resembling a real person. Individuals with ASD worked in pairs and played dual roles in mock job interviews. Specifically, one participant acted as the interviewee, while the other participant operated the android robot and acted as the interviewer. Eight individuals with high-functioning ASD participated in this study. After the training sessions, participants learned to understand the point of view of the interviewer, which may contribute to increased recognition of the importance of gestures and the motivation to learn how to behave in a job interview. In addition, participants reported improved self-confidence. These results provide preliminary support for the efficacy of playing dual roles using android robots.

## Introduction

Autism spectrum disorders (ASD) are lifelong conditions that impact many aspects of life, including employment outcomes. The low employment rate of individuals with ASD has become a major societal concern worldwide (1). Individuals with ASD have verbal and nonverbal social communication deﬁcits that can interfere with the reciprocity and ﬂow of conversation (2). Nonverbal communications, such as eye contact, facial expressions, and gestures, are assumed to be directly connected to poor performance during job interviews (3, 4). For example, certain nonverbal mistakes (e.g., individuals with ASD not looking at the interviewer in the eye and not making adequate facial expressions) can decrease the chances of receiving a job offer, even if the answers to the interview questions are impressive.

Recently, an advanced project using internet-accessed training and virtual reality has demonstrated that these tools have partial efficacy in improving job interview skills in individuals with ASD. For example, Strickland et al. showed that individuals with ASD who completed the internet-accessed training program had signiﬁcantly improved verbal communication skills compared to a control group (3). Burke et al. used a Virtual Interactive Training Agent system and showed that individuals with ASD develop the ability to identify strengths, self-promote, self-advocate, and answer situational questions (5). However, these studies did not show improvements in nonverbal communication.

In the job interview setting, nonverbal communication is at least as important as verbal communication is. According to a previous study, 55% of the first impressions are based on nonverbal communication, and only 7% of the first impressions are based on the actual verbal content of the interview (6). Thus, novel interventions for improving nonverbal communication skills are needed.

Unfortunately, as previously reported, improving nonverbal communication skills is more difficult than improving verbal communication skills (3). Challenges in this area are believed to occur because individuals with ASD are impaired in their ability to recognize other’s perspectives (7); that is, they lack what has been labeled the theory of mind (ToM) (8). Therefore, they are unable to understand the effect of their behavior on others (3), which is associated with their low motivation to acquire nonverbal communication skills (9). In designing an intervention to help individuals with ASD to acquire nonverbal communication skills, it is important to not only teach the appropriate nonverbal skills required for such an interaction (e.g., making eye contact and using appropriate facial expressions) but also improve their understanding of the importance of nonverbal communication. However, interventions for job interview preparation derived from a ToM viewpoint are limited (10).

To deepen the understanding of the point of view of interviewers, we developed a role-play-based guidance system for job interviews using an android robot, which is a robot with the appearance and movements resembling those of an actual human. In this system, participants were grouped in pairs and played dual roles using the android robot (i.e., the interviewee in a mock job interview by facing the android robot and the interviewer by operating the android robot). A comparison of the method used in this study and that in a previous study (11) shows that the intervention targets are distinct, because in the present study, the intervention involved learning to understand the point of view of the interviewer, while in the previous study, it involved simple exposure to the android robot-mediated interview procedures.

Greater self-confidence is important in tackling the target situation (12). Recent work has suggested the importance of measuring self-report in individuals with ASD (11, 13, 14). The primary objective of this study was to test whether our role-play-based-guidance system is useful for improving the self-confidence of individuals with ASD in a job interview. Second, we tested whether individuals with ASD show improvement in the recognition of the importance of gestures, motivation, and the extent of understanding the point of view of the interviewers. We hypothesized that after receiving our role-play-based-guidance, participants would demonstrate better self-confidence and show improvement in the recognition of the importance of gestures, motivation, and the extent of understanding the point of view of the interviewers.

## Material and Methods

### Participants

All procedures involving human participants were conducted in accordance with the ethical standards of the institutional and/or national research committee and with the 1964 Helsinki Declaration and its later amendments or comparable ethical standards. The present study was approved by the ethics committee of Kanazawa University. After receiving a complete explanation of the study, all participants and their guardians agreed to participate in the study. All participants provided written informed consent. Inclusion criteria included (1) diagnosis of ASD based on the *Diagnostic and Statistical Manual of Mental Disorders, Fifth Edition* (*DSM-5*) from the supervising study psychiatrist (15); (2) aged 19–25 years; and (3) unemployed workers who were actively seeking employment. All participants had known each other for at least 1 year. At the time of enrollment, the diagnoses of all participants were confirmed by a psychiatrist with more than 10 years of experience in ASD using the criteria in the *DSM-5* (15) and standardized criteria in the Diagnostic Interview for Social and Communication Disorders (DISCO) (16). The DISCO is reported to have good psychometric properties (17). All participants were diagnosed with Asperger’s syndrome according to the DISCO.

All participants completed the Autism Spectrum Quotient–Japanese version (AQ-J) (18), which was used to evaluate ASD-specific behaviors and symptoms. The AQ-J is a short questionnaire with five subscales (social skills, attention switching, attention to detail, imagination, and communication). Previous studies using the AQ-J have been replicated across cultures (19) and age (20, 21). The AQ is sensitive to the broader autism phenotype (22). Intelligence quotient (IQ) was measured by either the Wechsler Intelligence Scale for Children–Fourth Edition or the Wechsler Adult Intelligence Scale–Third Edition.

Despite treatments such as social skill training and behavioral therapy to improve communication skills and mock job interview training by humans, they could not perform standard nonverbal behaviors required for job interviews (e.g., making eye contact and facial expression). They had failed job interviews more than 10 times. We administered questionnaires pertaining to confidence and avoidance of job interviews, in which items were rated on a 7-point Likert scale half a year before the experiment, and confirmed that all participants had not been confident and had avoided attending job interviews half a year before. The ratings ranged from 1 (not at all comfortable) to 7 (very comfortable). The first author conducted a semistructured interview question, in which participants were asked, “Do you think nonverbal behavior is important in a job interview?” All participants answered that nonverbal behavior is not important in a job interview. They seemed to have a similar level of understanding in that they considered nonverbal behavior to be not important in a job interview.

### Procedures

**Figure 1:**
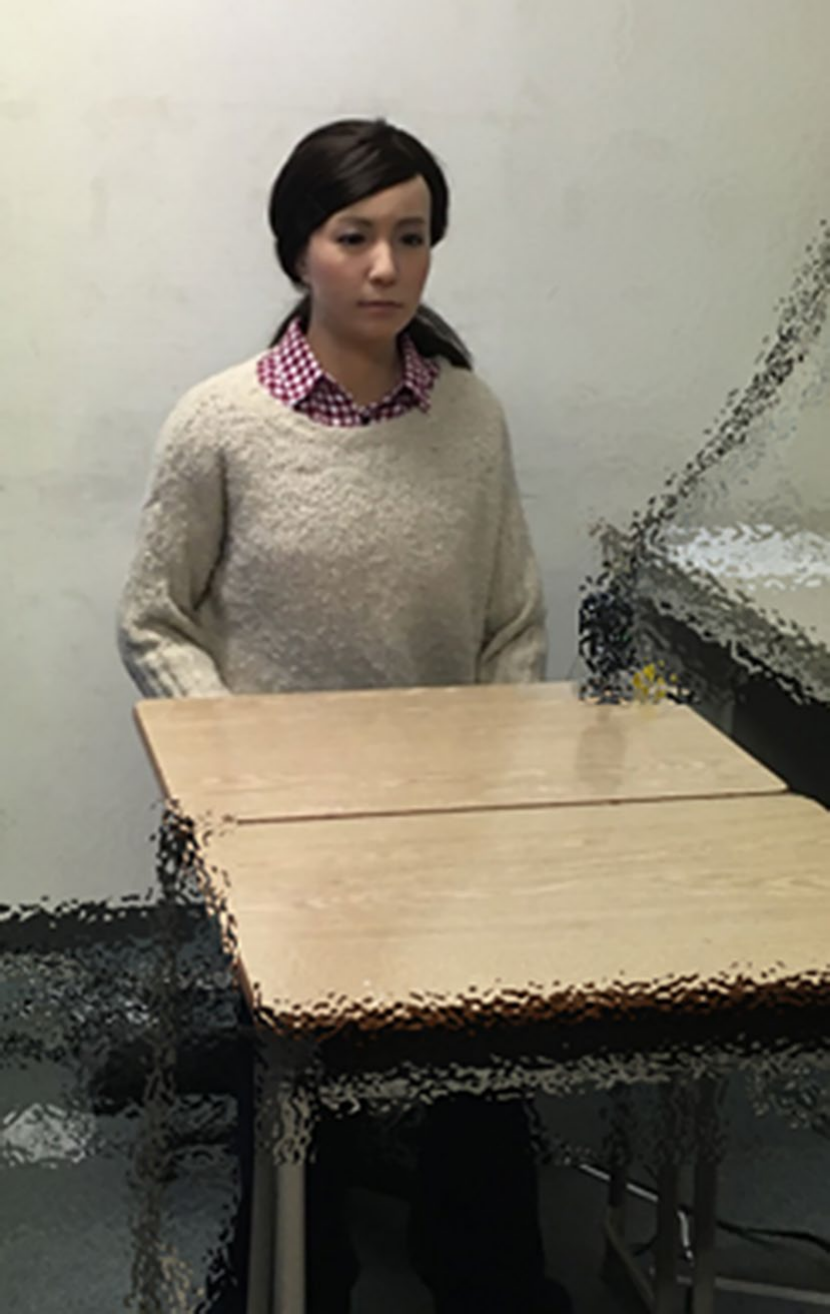
Actroid-F (android robot).

The android robot used in this study was the Actroid-F (14, 23, 24) (**Figure 1**) (Kokoro Co. Ltd. Hamura, Tokyo, Japan), which is approximately 165 cm in height and is a female humanoid robot. The face was made of soft silicon rubber by taking a copy of a real human face. It has 12 degrees of freedom in the upper body as follows: eyebrows (up/down, wrinkling); eyelids (open/close); eyeballs (pan, tilt); mouth (open/close, smiling); head (turn, nod, lean); chest (breathing); and waist (bowing). Actroid-F’s face can show a range of simplified expressions, but in a less complex way compared to a real human face. Its artificial body has the same proportions, facial features, hair color, and hairstyle as a human. In this experiment, the Actroid-F was remotely teleoperated by the interviewer (i.e., one of the participants).

On days 1 and 6, the participants participated in a 10-min mock job interview with a human confederate for basic evaluation. Initially, participants were given a document containing recruitment information from which they could select a job including data entry clerk, shelf stacker in a supermarket, custodian, kitchen assistant in a restaurant, nursing assistant, and paper delivery person. After the mock job interviews, we asked participants to complete a questionnaire about their self-confidence in their interview performance. Items were rated on a 7-point Likert scale (ranging from 1 = “not at all comfortable” to 7 = “very comfortable”). We asked participants to complete a questionnaire containing a 5-point Likert scale (1–5) for the question, “How important do you think are gestures in a job interview?” Responses ranged from 1 (I cannot understand the importance of gestures in a job interview at all) to 5 (I understand the importance of gestures in a job interview perfectly). We also asked participants to complete a questionnaire containing a 5-point Likert scale (1–5) for the question, “How motivated are you to learn how to perform in job interviews?” Responses ranged from 1 (I am not motivated to learn how to perform in job interviews at all) to 5 (I am very motivated to learn how to perform in job interviews). In addition, the first author conducted semistructured interviews about “others’ viewpoint.” The participants were asked two questions. First, to assess whether each participant knew that their perspective is different from most people’s perspective, the first author asked, “Do you think your perspective is different from most people’s?” The first author then asked, “Please rate, on a scale from 1 (I cannot understand the point of view of interviewers at all in a job interview setting) to 5 (In a job interview setting, I can understand the point of view of interviewers perfectly), the extent to which you understand the point of view of the interviewers.” After the intervention, the first author asked their teacher, “Did the students learn to understand the point of view of the interviewer after the intervention?”

**Figure 2:**
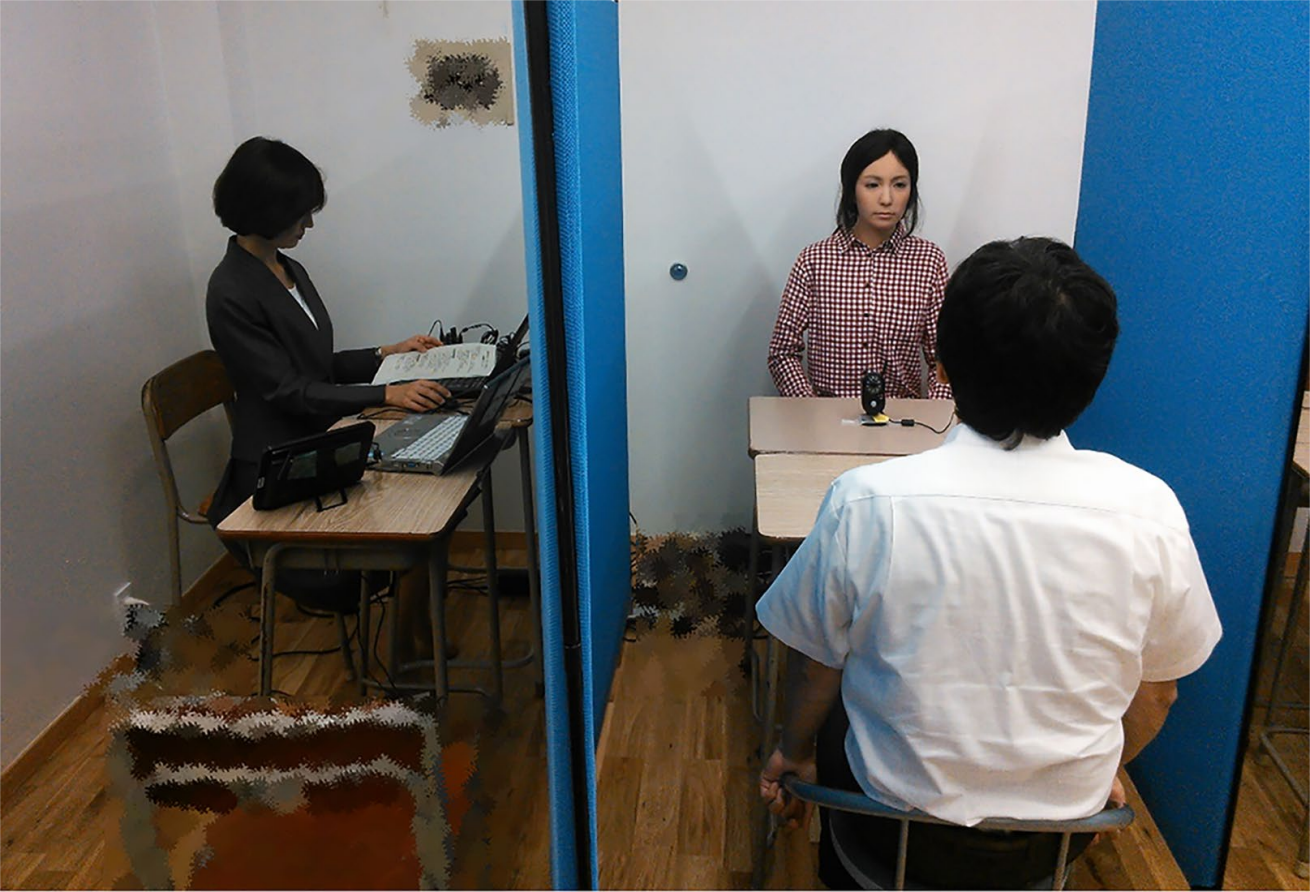
The person on the left of the figure played the role of the interviewer by teleoperating the Actroid-F. At the right back of the room is the Actroid-F. The person facing the Actroid-F played the role of the interviewee.

From days 2 to 5, all eight individuals were grouped in pairs and participated in the mock interviews at the same time. One participant acted as the interviewee in the mock job interview by facing an Actroid-F that was remotely controlled by the other participant, who acted as the interviewer (**Figure 2**). The other participant (i.e., interviewer) asked questions by selecting from the script (see **Supplementary Material**), which we prepared in advance. Buttons that corresponded to each script were displayed on the monitor, and the interviewer could ask a question by pushing a button, which was read out loud by the Actroid-F. The interviewer could also replicate facial expressions, such as smile, surprise, or sorrow, by using the button. The interviewer could monitor the expressions made by the Actroid-F and the interlocutor *via* a video. They practiced 20 min every day (i.e., they practiced each role for approximately 10 min). Considering their interest in operating the android robot, we suggested that playing the role of the interviewer by operating an android robot is useful to learn the point of view of an interviewer in a job interviewer setting. For many individuals with ASD, sensory overstimulation is a serious problem (25), and the flood of social cues may be a primary cause for the inability to process social signals. The burden of getting information from the interlocutor through the monitor is lower than getting information from the interlocutor directly in terms of sensory stimulation. This facilitates information processing even for individuals with ASD and helps in understanding another person’s perspective (24). Controlling their facial expression and conversation freely by typing on a keyboard is easier for them than speaking face to face, which reduces the burden in speaking and contributes to directing their energy toward information processing (24). In addition, for all participants, an unfamiliar person is difficult to approach, so being paired with a familiar person supports a smooth interaction.

We conducted a brief survey and interview with all participants from this study to evaluate their vocational outcomes at 1-year follow-up with a focus on whether or not they attained a competitive position. The interview included questions about whether the experience of playing dual roles using the android robot was the trigger to put themselves in the position of the interviewer.

### Statistical Analysis

All statistical analyses were performed using IBM SPSS Statistics 24.0 (IBM, Armonk, NY, USA). To test the first hypothesis that our role-play-based guidance is useful for improving self-confidence in job interviews, a paired *t*-test was performed to evaluate the ratings of self-confidence between days 1 and 6. To test the second hypothesis that our role-play-based guidance is useful for improving the recognition of the importance of gestures, motivation, and the extent of understanding the point of view of interviewers, a paired *t*-test was also performed between days 1 and 6.

## Results

### Feasibility and Participation

In total, eight individuals with ASD took part in the study (see **Table 1** for participant details). All participants completed the trial procedures without technological challenges or noted participant distress that would lead to session termination. We carefully observed participant performance and confirmed that all participants were concentrating during the trials and highly motivated from the start to the finish of the experiment.

**Table 1:** Descriptive statistics of participants.

Characteristics	*n* = 8 M (SD)
Age (years)	22.5 (2.2)
Gender (males: female)	8:0
Full scale IQ	83.1 (9.7)
AQ-J	30.3 (4.1)

M, mean; SD, standard deviation; AQ-J, Autism Spectrum Quotient-Japanese version.

### Primary Analyses

All participants operated with the Actroid-F with enthusiasm evidenced by full participation and comments indicating that they enjoyed the process. When acting as the interviewee, participants seemed to be focused and engaged in the mock job interview. During the intervention, they learned the importance of gestures in job interviews from their experience as the interviewer, and these experiences increased their motivation to learn how to perform in job interviews. There was also a significant increase in the recognition of the importance of gestures in a job interview on day 6 (3.75 ± 0.71) compared to day 1 (1.63 ± 0.74) (*p* < 0.01) (see **Table 2**). There was a significant increase in the motivation to learn how to perform in job interviews on day 6 (4.25 ± 0.46) compared to day 1 (2.25 ± 0.71) (*p* < 0.01) (see **Table 2**). This motivation led to a positive attitude toward the role of the interviewee in the mock job interview training. These experiences seemed to increase participants’ self-confidence. In fact, there was a significant increase in self-confidence on day 6 (4.50 ± 0.93) compared to day 1 (2.50 ± 1.07) (*p* < 0.01) (see **Table 2**). Following the intervention, we confirmed by a semistructured interview that all participants learned to understand the point of view of the interviewer during a job interview. Moreover, in response to the first interview question on day 1, all participants answered, “I understand my perspective is different from most people’s.” On day 6, six of the eight participants answered, “By participating in this study, I truly realized that my perspective is different from most people’s.” The result of the second interview question indicated a significant improvement in understanding the point of view of the interviewer on day 6 (3.38 ± 0.74) compared to day 1 (1.38 ± 0.52) (*p* < 0.01) (see **Table 2**). In the semistructured interview, their teachers answered, “All students seemed to learn to understand the point of view of the interviewer.” They started thinking about job interviews positively and restarted their job searches. At the 1-year follow-up after the intervention, five participants had passed job interview examinations and found employment. In our interview 1 year after the intervention, all participants answered, “The ex­perience of role-play-based guidance using the android robot was the trigger to put ourselves in the position of the interviewer.”

**Table 2:** Descriptive statistics of participants.

Outcome	Baseline M (SD)	Post-intervention M (SD)	*t*	Statistics *F*	*p*
Self-confidence	2.50 (1.07)	4.50 (0.93)	–10.583	7	<0.01**
Recognition of the importance of gestures	1.63 (0.74)	3.75 (0.71)	–4.432	7	<0.01**
Motivation	2.25 (0.71)	4.25 (0.46)	–6.110	7	<0.01**
The extent of understanding the point of view of interviewers	1.38 (0.52)	3.38 (0.74)	–4.733	7	<0.01**

M, mean; SD, standard deviation.

**p < 0.01.

## Discussion

In this study, by playing the dual roles using the Actroid-F, the experience of playing the interviewer *via* the android robot increased participants’ recognition of gestures being a crucial aspect of communication and the importance of knowing how to perform in a job interview. Participants learned to understand the point of view of the interviewer, which may have contributed to the increased recognition of the importance of gestures and the motivation to learn how to behave in a job interview. In general, individuals with ASD have difficulty recognizing that their perspectives differ from those of most people. Therefore, a lack of recognition is indicative of a lack of ToM, which may be the cause of many behaviors associated with ASD, such as inappropriate facial expression and eye contact. Owing to the lack of this insight, individuals with ASD cannot understand the effects of their behaviors on others (2). That is, our system may help alleviate the ToM deficit of individuals with ASD in job interview scenarios.

Placement in pairs of individuals who knew each other also seemed to contribute to their understanding of the perspective of the interviewer. In addition, in this system, acting as the interviewer using the Actroid-F has many advantages compared to conversing face to face. Specifically, when conversing face to face, sensory overstimulation from the human interviewee is a serious problem for individuals with ASD, and it interferes with the processing of social signals (26). Furthermore, the technology behind the Actroid-F might increase the user’s enthusiasm for and concentration on the intervention. In addition, acting as the interviewee by facing the Actroid-F may have the advantage of decreasing interpersonal anxiety and promote intrinsic motivation for the mock job interview. These mechanisms might have enriched the participants’ understanding and led to improvements in their self-confidence.

Young adults with ASD are able to self-report psychiatric symptoms (26). Compared to their caregivers, they may be more accurate reporters of their own mood dysregulation (27). Considering these factors, the results of the self-reporting questionnaire on self-confidence and motivation in this study (participants improved their self-confidence in interview performance and motivation to learn how to perform job interview) are reliable.

In this study, based on semistructured interviews with participants and their teachers, our intervention indicates improved perspective-taking in participants. Considering the usefulness of double scoring by participants and their teachers (26), the results of the semistructured interview are reliable. Furthermore, the participants demonstrating an increased recognition of the importance of gestures and motivation to learn how to behave in a job interview also indicates that participants gained a deeper understanding of taking on the perspective of another, an important component of the ToM.

The experience of playing dual roles using the android robot seems to help participants understand the position of the interviewer. However, it is difficult to conclude that a daily 20-min intervention for 6 days is sufficient to explain the long-term outcome (i.e., five participants had passed a job interview examination and found employment). The existence of confounding variables, such as interventions and education that participants received after the experiment, makes it difficult to establish a clear link between the intervention and the outcome. Future studies controlling for the confounding factors are needed.

This study has several limitations. The first limitation is the relatively small number of participants. Larger sample sizes are necessary to provide more meaningful data to evaluate the efficacy of role-play-based guidance system using an android robot. Second, in addition to the self-reporting and the interviews with the participants, we asked their teacher to confirm whether they had learned to understand the point of view of the interviewer. We also confirmed at the 1-year follow-up after the intervention that five of the eight participants had passed their job interviews and found employment. On the other hand, we did not measure biological markers, which is a limitation of this study. Although young adults with ASD may be accurate reporters of their own mood dysregulation compared to their caregivers (27), future studies measuring not only self-report but also biological markers, such as saliva cortisol, are needed.

In conclusion, as hypothesized, individuals with ASD demonstrated improved recognition of the importance of gestures, motivation, and the extent of understanding the point of view of interviewers. In addition, they demonstrated better self-confidence after receiving our role-play-based guidance using the android robot. While robotic technologies are considered potential vehicles for enhancing nonverbal communication skills in children with ASD, a few studies have been using this strategy to investigate the ability of children with ASD to recognize others’ perspectives. The present study provides preliminary support for a unique application of a robotic system (e.g., role-play based guidance system using android robot) to overcome a component (e.g., job interviewing) of a very specific challenge (e.g., employment) that many individuals with ASD struggle with over time. The system used in this study is not personalized according to their needs. To personalize this system according to individuals’ different needs, future developments of the system to adjust the intelligibility of nonverbal behaviors for android robot depending on the situation are needed.

## Ethics Statement

All procedures involving human participants were conducted in accordance with the ethical standards of the institutional and/or national research committee and with the 1964 Helsinki Declaration and its later amendments or comparable ethical standards. After an explanation of the study was provided, all participants’ guardians provided written, informed consent.

## Author Contributions

HK designed the study, conducted the experiment, carried out the statistical analyses, analyzed and interpreted data, and drafted the manuscript. TM, YY, YM, HI, MM, and MK conceived of the study and participated in its design and assisted with data collection and scoring of behavioral measures and analyzed and interpreted the data and were involved in drafting the manuscript and revised it critically for important intellectual content. MK was involved in approving the final version to be published. All authors read and approved the final manuscript.

## Funding

This work was supported in part by Grants-in-Aid for Scientific Research from the Japan Society for the Promotion of Science (17H05857), ERATO ISHIGURO Symbiotic Human-Robot Interaction Project, and was partially supported by The Center of Innovation Program from the Japan Science and Technology Agency, JST, Japan.

## Conflict of Interest Statement

The authors declare that the research was conducted in the absence of any commercial or financial relationships that could be construed as a potential conflict of interest.

## References

[1] Volkmar FR State M Klin A Autism and autism spectrum disorders: diagnostic issues for the coming decade. J Child Psychol Psychiatry (2009) 50:108–15. 10.1111/j.1469-7610.2008.02010.x 19220594

[2] Morgan L Leatzow A Clark S Siller M Interview skills for adults with autism spectrum disorder: a pilot randomized controlled trial. J Autism Dev Disord (2014) 44:2290–300. 10.1007/s10803-014-2100-3 24682707

[3] Strickland DC Coles CD Southern LB JobTIPS: a transition to employment program for individuals with autism spectrum disorders. J Autism Dev Disord (2013) 43:2472–83. 10.1007/s10803-013-1800-4 PMC3706489 23494559

[4] Kandalaft MR Didehbani N Krawczyk DC Allen TT Chapman SB Virtual reality social cognition training for young adults with high-functioning autism. J Autism Dev Disord (2012) 43:34–44. 10.1007/s10803-012-1544-6 PMC3536992 22570145

[5] Burke SL Bresnahan T Li T Epnere Rizzo Partin Using virtual interactive training agents (ViTA) with adults with autism and other developmental disabilities. J Autism Dev Disord (2018) 48:905–12. 10.1007/s10803-017-3374-z 29168090

[6] Albert M Silent messages: implicit communication of emotions and attitudes. Belmont: Wadsworth Pub. Co. (1971).

[7] Baron-Cohen S Tager-Fulsberg H Cohen DJ Understanding other minds: perspectives from developmental cognitive neuroscience. Oxford: Oxford University Press (2000).

[8] Baron-Cohen S Leslie AM Frith U Does the autistic child have a “theory of mind”? Cognition (1985) 21:37–46. 10.1016/0010-0277(85)90022-8 2934210

[9] Chevallier C Kohls G Troiani V Brodkin ES Schultze RT The social motivation theory of autism. Trends Cogn Sci (2012) 16:231–9. 10.1016/j.tics.2012.02.007 PMC3329932 22425667

[10] Begeer S Howlin P Hoddenbach E Clauser C Lindauer R Clifford P Effects and moderators of a short theory of mind intervention for children with autism spectrum disorder: a randomized controlled trial. Autism Res (2015) 8:738–48. 10.1002/aur.1489 25847054

[11] Kumazaki H Warren Z Corbett BA Yoshikawa Y Matsumoto Y Higashida H Android robot-mediated mock job interview sessions for young adults with autism spectrum disorder: a pilot study. Front Psychiatry (2017) 8:169. 10.3389/fpsyt.2017.00169 28955254 PMC5601082

[12] Maskey M Lowry J Rodgers J McConachie H Parr JR Reducing specific phobia/fear in young people with autism spectrum disorders (ASDs) through a virtual reality environment intervention. PLoS One (2014) 9:e100374. 10.1371/journal.pone.0100374 24987957 PMC4079659

[13] Mikita N Hollocks MJ Papadopoulos AS Aslani A Harrison S Leibenluft E Irritability in boys with autism spectrum disorders: an investigation of physiological reactivity. J Child Psychol Psychiatry (2015) 56:1118–26. 10.1111/jcpp.12382 PMC4737220 25626926

[14] Simon DM Corbett BA Examining associations between anxiety and cortisol in high functioning male children with autism. J Neurodev Disord (2013) 5:32. 10.1186/1866-1955-5-32 24216056 PMC3827503

[15] American Psychiatric Association (APA) Diagnostic and statistical manual of mental disorders. 5th ed Arlington, VA: American Psychiatric Publishing (2013) p. 5–25.

[16] Leekam SR Libby SJ Wing L Gould J Taylor C The diagnostic interview for social and communication disorders: algorithms for ICD-10 childhood autism and wing and gould autistic spectrum disorder. J Child Psychol Psychiatry (2002) 43:327–42. 10.1111/1469-7610.00024 11944875

[17] Wing L Leekam SR Libby SJ Gould J Larcombe M The diagnostic interview for social and communication disorders: background, inter-rater reliability and clinical use. J Child Psychol Psychiatry (2002) 43:307–25. 10.1111/1469-7610.00023 11944874

[18] Wakabayashi A Tojo Y Baron-Cohen S Wheelwright S [The Autism-Spectrum Quotient (AQ) Japanese version: evidence from high-functioning clinical group and normal adults]. Shinrigaku Kenkyu (2004) 75:78–84. 10.4992/jjpsy.75.78 15724518

[19] Wakabayashi A Baron-Cohen S Uchiyama T Yoshida Y Tojo Y Kuroda M The autism-spectrum quotient (AQ) children’s version in Japan: a cross-cultural comparison. J Autism Dev Disord (2007) 37:491–500. 10.1007/s10803-006-0181-3 16944324

[20] Auyeung B Baron-Cohen S Wheelwright S Allison C The Autism Spectrum Quotient: Children’s Version (AQ-Child). J Autism Dev Disord (2008) 38:1230–40. 10.1007/s10803-007-0504-z 18064550

[21] Baron-Cohen S Hoekstra RA Knickmeyer R Wheelwright S The Autism-Spectrum Quotient (AQ)—adolescent version. J Autism Dev Disord (2006) 36:343–50. 10.1007/s10803-006-0073-6 16552625

[22] Wheelwright S Auyeung B Allison C Baron-Cohen S Defining the broader, medium and narrow autism phenotype among parents using the Autism Spectrum Quotient (AQ). Mol Autism (2010) 1:10. 10.1186/2040-2392-1-10 20678260 PMC2913943

[23] Yoshikawa M Matsumoto Y Sumitani M Ishiguro H Development of an android robot for psychological support in medical and welfare fields. 2011 IEEE International Conference on Robotics and Biomimetics, Phuket, Thailand (2011) 2378–83. 10.1109/robio.2011.6181654

[24] Johnson CP Myers SM American Academy of Pediatrics Council on Children With D. Identification and evaluation of children with autism spectrum disorders. Pediatrics (2007) 120:1183–215. 10.1542/peds.2007-2361 17967920

[25] Kumazaki H Muramatsu T Yoshikawa Y Matsumoto Y Miyao M Ishiguro H Tele-operating an android robot to promote the understanding of facial expressions and to increase facial expressivity in individuals with autism spectrum disorder. Am J Psychiatry (2017) 174:904–5. 10.1176/appi.ajp.2017.17030257 28859509

[26] Hurtig T Kuusikko S Mattila M-L Haapsamo H Ebeling H Jussila K Multi-informant reports of psychiatric symptoms among high-functioning adolescents with Asperger syndrome or autism. Autism (2009) 13:583–98. 10.1177/1362361309335719 19933765

[27] Schupp CW Simon D Corbett BA Cortisol responsivity differences in children with autism spectrum disorders during free and cooperative play. J Autism Dev Disord (2013) 43:2405–17. 10.1007/s10803-013-1790-2 PMC3885342 23430177

