# Supplementary material for: Role-Play-Based Guidance for Job Interviews Using an Android Robot for Individuals With Autism Spectrum Disorders
Source: Front Psychiatry. 2019 Apr 11;10:239. doi: 10.3389/fpsyt.2019.00239 (PMC6470286; doi:10.3389/fpsyt.2019.00239)
Supplement: Supplementary file 1 [file DataSheet_1.docx]

**Supplementary material**

The dialogue between the participant and the interviewer (the android robot or the human

interviewer) in the mock interview was partially structured by using the following script in this

supplementary material. The sentences listed in (1), (2) and (3) were used as the utterance of the

interviewer. The interviewer basically uttered a sentence in the list (1) as a question, waiting for

the response from the participant, and said either sentence in the list (2) as the response to each

question. The utterance of the list (3) were sometimes inserted between the question and

the acknowledgement to let the theme dig more deeply. The questions were given in the

numerical order in the list (1) in the all days of the experiment while different two questions

were chosen to be dug into in each day of the experiment.

(1) Examples of scripts for mock job interview

1. Please take a seat.
2. Good afternoon. Hello there.
3. Thank you for applying, my company XX.
4. Well then, could you introduce yourself?
5. Would you please tell me the reasons of your application?
6. Why would you like to get a job? Please tell me.
7. What are the things you are good at?

What is your special talent?

1. What are the things you are not good at?
2. How are you coping with the things you are weak in?
3. What kind of things would you like to do in our company?

Please tell me what you can do.

1. What are you doing in the vocational training school? From what time until what time is it?
2. Have you ever failed at your work?
3. Please explain your disability briefly.
4. How are you feeling now?
5. In my company, we have a lot of jobs that need standing. Is that all right?
6. Is it fine with you to carry heavy stuff?
7. Are you on any medicine now?
8. Is there anything you would like us to consider?
9. Which route did you take to come here from your home?
10. Are there any working days or working hours you particularly wish?
11. Is there anything else you would like to talk to us?
12. I understand. Thank you very much.

(2) The words prepared as responses (to make the conversation go smoothly)

・ Yes

・ Certainly

・ All right

・ Very well

・ Thank you

・ I see. Now I understand.

・ Oh, I see. I got it.

・ I see. That makes sense.

・ Great!

・ Wonderful!

・ Fantastic.

・ Incredible.

(3) Words in order to dig into further

・ Please tell me a bit more in detail.

・　Why do you think so?
